# Membrane-modified mesoporous silica nanoparticles guided by tumor immunomodulatory regulation for anti-tumor strategies

**DOI:** 10.3389/fimmu.2026.1796382

**Published:** 2026-04-22

**Authors:** Yixuan Gao, Haonan Wu, Tingting Liu, Xueyu Pu, Tangjun Ren, Chen An, Han Zhang, Jian Yang

**Affiliations:** 1 State Key Laboratory of Chinese Medicine Modernization, Tianjin University of Traditional Chinese Medicine, Tianjin, China; 2 Institute of Traditional Chinese Medicine, Tianjin University of Traditional Chinese Medicine, Tianjin, China; 3 Department of Colorectal Surgery, Tianjin Union Medical Center, The First Affiliated Hospital of Nankai University, Tianjin, China

**Keywords:** cancer therapy, cell membrane, drug delivery system, mesoporous silica nanoparticles, tumor immune microenvironment

## Abstract

The effective treatment of cancer remains challenging due to the highly immunosuppressive and complex tumor immune microenvironment (TIME). Mesoporous silica nanoparticles (MSNs), known for their tunable pore structures and high drug-loading capacity, have been extensively employed in cancer diagnosis and therapy. To combat the development of cancer, emerging studies on cell membrane-coated MSNs (CM-MSNs) reveal that these systems integrate the intrinsic features of MSNs with unique biological functions inherited from source cells—such as immune evasion, tumor targeting, and prolonged circulation—thereby addressing key limitations of bare nanoparticles, including poor targeting efficiency and uncontrolled drug release. As a biomimetic drug delivery platform, they enhance tumor-targeted accumulation through membrane-mediated homing and exert profound immunomodulatory effects within TIME. This review systematically summarizes recent advances in MSNs coated with membranes derived from diverse cellular sources, including innate and adaptive immune cells, blood cells, cancer cells, and engineered hybrid cells, with a particular focus on their roles in regulating the tumor–immune interface. We further discuss the prospects and challenges for the clinical translation of CM-MSNs. Collectively, these developments inspire innovative designs for biomimetic nanoplatforms and open new avenues for optimizing cancer immunotherapy, both via precise manipulation of cellular interactions at the immune interface.

## 1 Introduction

As one of the major public health problems in the world, cancer has high morbidity and mortality, leading to an increasing burden of cancer treatment (1, 2). According to the World Health Organization, over 35 million new cancer cases are predicted in 2050, a 77% increase from the estimated 20 million cases in 2022 (3). The traditional treatment strategies such as surgery, chemotherapy, and radiation therapy have been developed to suppress tumor cell growth, but they are often limited by their insufficient local drug concentrations, rapid clearance, and accidental toxic side effects, leading to patient compliance reduction (4, 5).

To overcome these limitations, nanomedicine has advanced rapidly, and various nanoparticles have been widely used for precise cancer therapy (6). Because of the uniform pores and easy surface functionalization, MSNs are commonly used in tumor diagnosis and treatment (7). These intrinsic structural and chemical merits grant MSNs exceptional engineering application potential in oncology, allowing for the rational design and precise nanoscale engineering of multifunctional theranostic nanoplatforms to fulfill the unmet needs of precision cancer management, which has been widely validated in preclinical studies covering structural modulation, functional customization and clinical translation-oriented development of MSNs (8–10). Decorating with biological molecules or polymer moieties on the surface of MSNs can achieve functions such as controlling drug release profile, improving drug loading capacity, reducing drug toxicity, and enhancing targeting ability (11, 12). However, these functionalized MSNs still face challenges such as low targeting efficacy, immune recognition, and a short half-life, which collectively result in suboptimal delivery efficiency (13). For instance, in multiple mouse tumor models, the targeting efficiency of trastuzumab and folic acid-modified MSNs to cancer cells was less than 0.0014% (14). Ishida et al. reported that polyethylene glycol exhibited unexpected immune reaction, which led to rapid clearance of a second dose (15). In addition, in the chemical modification process, it is usually necessary to use coupling agents or reactive ligands to modify the surface of nanoparticles, followed by complex purification. This process is inefficient and cumbersome, and is prone to introducing impurities or toxic reagents during the chemical reaction process (16, 17). Due to the complexity of biological environment, some performance such as long circulation time, immune evasion, and specific targeting cannot achieve satisfactory results through conventional modification, which limits the application of MSNs (18).

**Figure 1:**
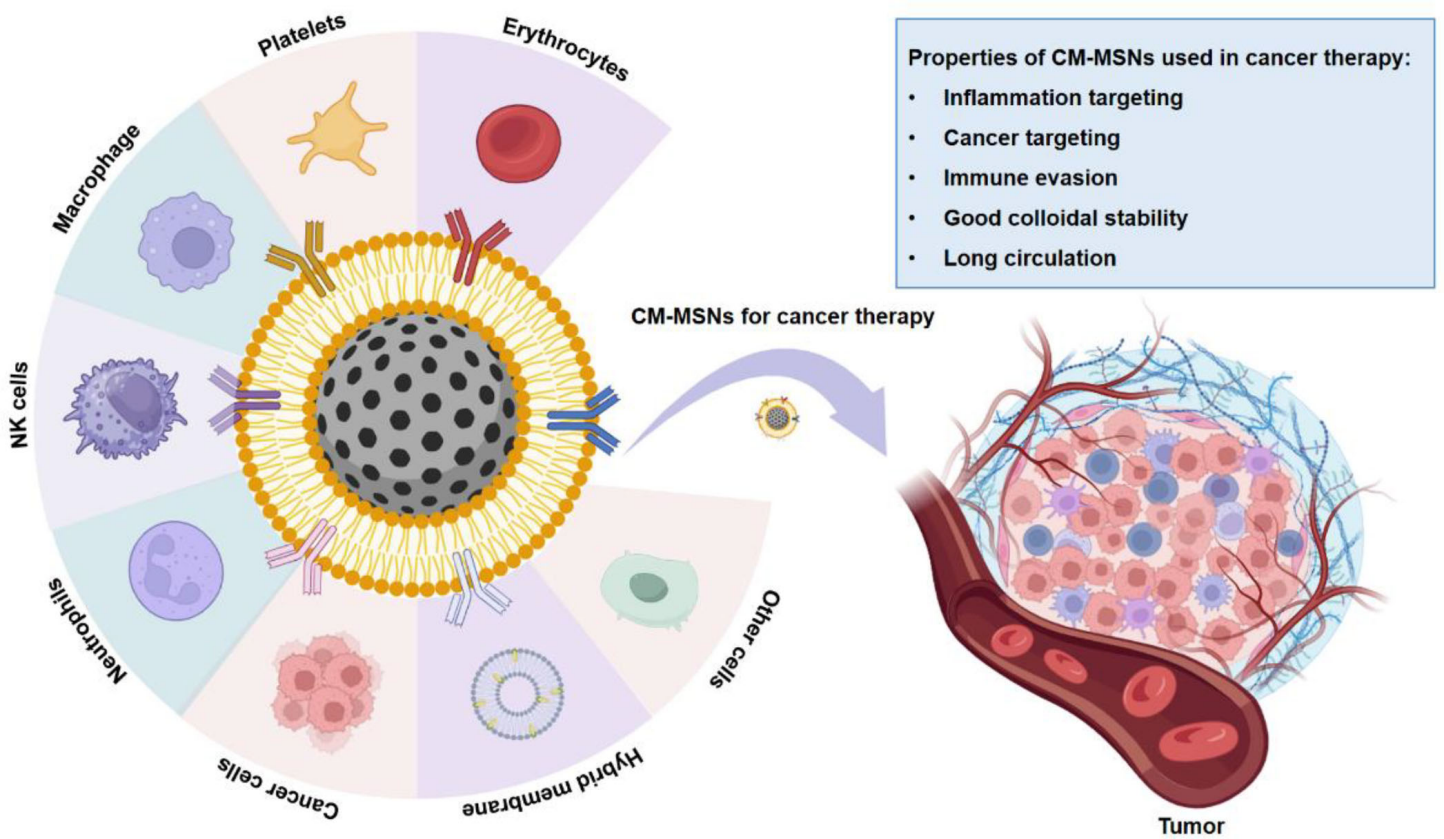
Mesoporous silica nanoparticles modified with cell membranes from diverse sources exhibit excellent stability, long circulation, immune evasion, tumor immune microenvironment targeting, and regulate the tumor immune interface for cancer therapy (image was created with BioRender.com).

Cell membrane coating technology enables nanoparticles to inherently replicate the biological properties of the source cells (19). While PEG modification alleviates protein corona, poor colloidal stability and immune clearance, it still causes accelerated blood clearance and systemic immunogenicity (20). In comparison, membrane-coated mesoporous silica nanoparticles avoid these defects and possess stable circulation, efficient immune evasion and intrinsic homologous targeting (21). Therefore, cell membranes derived from various cells like cancer cells, erythrocytes, and platelets, have been used to achieve functional diversification of MSNs and improve their delivery efficiency *in vivo* (22–24). For example, nanoparticles coated with cell membranes derived from macrophage, neutrophil, and natural killer cells possess the ability to respond to chemoattractant secreted by tumors and recruit to the tumor sites, instantly attacking antigens on tumor cells, thereby achieving cancer targeting (25). The biomimetic modification of cell membrane enables mesoporous silica nanoparticles to actively simulate and intervene in the natural membrane-membrane interaction, thus achieving accurate targeting, signal transmission and immune cell reprogramming at the tumor immune interface (26). Consequently, their interaction with biological membranes shifts from non-specific, pro-inflammatory foreign body recognition to a tolerant form of membrane interface communication based on specific molecular recognition, thereby enabling passive evasion of the immune system (27) (**Figure 1**).

Combining the advantages of synthetic nanomaterials and natural cell membranes to overcome the complex tumor microenvironment (TME) and combat cancers is a viable treatment strategy (28). In this review, we summarize recent advances in membrane modification strategies of MSNs via CM-MSNs and highlight the functional diversity of various CM-MSNs in regulating the tumor immune interface for cancer treatment (**Table 1**). Furthermore, this review aims to provide a concise overview of recently reported CM-MSNs and offer a deeper insight into the precise targeted strategies of CM-MSNs for modulating the tumor interface during cancer therapy.

**Table 1:** CM-MSNs for antitumor applications.

Cell types	Nanoparticles	Preparation method	Drugs	Administration route	Therapeutic strategy	Disease	Ref
Erythrocyte from whole blood	Magnetic MSNs	Extrusion	Hypocrellin	Intravenous	Stability; Long circulation; Magnetic targeting; PDT	Breast cancer	(22)
	MSNR	Extrusion	DOX and ICG	Cellular uptake	Reduced drug leakage; Reduced macrophage phagocytosis; Chemo-photothermal therapy	Breast cancer	(29)
	Hollow MSNs	Extrusion	Oxophilic radiometal ^89^Zr	Intravenous	Stability; Long circulation; Photodynamic therapy	Colorectal cancer	(30)
	Mesoporous silica shell layered upconversion nanoparticles	Extrusion	Chlorin e6 and DOX	Intravenous	Long circulation; Immune evasion; Chemo-photodynamic therapy	HCC	(31)
	MSNs	Sonication	ICG and microRNA-137	Intravenous	Reduced macrophage phagocytosis; Cancer targeting (RGD modification); Photothermal and gene therapy	Glioblastomas	(32)
	MSNs	Extrusion	DOX	Cellular uptake	Long circulation; Cancer targeting (biotin modification); Chemotherapy	Cervical cancer	(33)
	Hollow mesoporous organosilica nanoparticles	Stirring	DOX	Intravenous	Stability; Long circulation; Cancer targeting (MUC-1 receptor modification); Chemotherapy	Colon adenocarcinoma	(34)
Platelet from whole blood	Mesoporous silica shell covered bismuth nanorod	Sonication	/	Intravenous	Cancer targeting; Immune escape; PTT and radiotherapy	Breast cancer	(35)
	MSNs	Sonication and stirring	Combretastatin A4 and apatinib	Intravenous	Crossing vascular barriers; Immune evasion; Significant vascular disruption and efficient anti-angiogenesis for tumor eradication	HCC	(36)
	MSNs	Sonication	Tirapazamine and 5,6-dimethylxanthenone-4-acetic acid	Intraperitoneal	Crossing vascular barriers; Hypoxia-sensitive chemotherapy	Colon cancer	(37)
	MSNs	Sonication	Anti-PD-L1 antibody and sorafenib	Intravenous	Surgical site targeting; Immune evasion	Postoperative recurrence of HCC	(38)
	Hollow MSNs	Incubation	Anti-PD-L1 antibody and sorafenib	Intravenous	Special targeting of CTCs; Immunotherapy/chemotherapy	HCC	(39)
RAW264.7 cell lines	Mesoporous silica-layered NaErF 4 @NaLuF 4 upconverting NPs co-doped with PFC/Ce6	Sonication	Paclitaxel, chlorine 6 and perfluorocarbon	Cellular uptake	Targeting to TAMs; Repolarizing TAMs to M1; TAM-mediated antitumor therapy	Breast cancer	(40)
	MSNs	Sonication	DOX, R848 and catalase	Cellular uptake	Reduced drug leakage; Immune evasion; Cancer targeting; Immunotherapy	HCC; Colon cancer	(41)
	FA modified MSNs with rod-like shape	Extrusion	DOX and ICG	Intravenous	Deep tumor penetration; Cancer targeting; Immune evasion; PTT, PDT and chemotherapy	Breast cancer	(42)
Neutrophil from whole blood	MSNs	Extrusion	Shanzhiside methylester and DOX	Intravenous	Cancer targeting; Reduced drug leakage; Chemotherapy and anti-inflammatory therapy	Lymphoma	(43)
Primary human T cells from peripheral blood	MSNs	Sonication and extrusion	IR780	Intravenous	Specifically recognizing GPC3 + HCC; Photothermal therapy	HCC	(44)
NK cells from mice spleen	Hollow mesoporous disulfide-bridged organosilica	Sonication and extrusion	Thermal azoinitiator AIPH	Intravenous	Immune evasion; Amplified cellular uptake; Thermodynamic-chemodynamic therapy	HCC	(45)
LNCaP-AI cell line	CaCO_3_ layered MSNs	Sonication	DOX	Intravenous	Homologous targeting; Chemotherapy	Prostate cancer	(46)
MCF-7 cell line	Carbon@silica with semi-yolk@spiky-shell structure	Sonication	DOX	Cellular uptake	Homologous targeting; Deep tumor penetration; Photo-chemotherapy	Breast cancer	(47)
B16-F10 cell line	MSNs	Extrusion	Glucose oxidase	Intravenous	Homologous targeting; Combining immunotherapy and starvation therapy	Melanoma	(48)
4T1 cell line	Copper sulfide NPs deposited dendritic MSNs	Sonication	R848	Intravenous	Homologous targeting; Combining photothermal ablation and immune remodeling	TNBC	(49)
Erythrocyte/U251 cancer cell	MSNs	Sonication	Gboxin	Intravenous	Homologous targeting; Chemotherapy	Glioblastomas	(50)
Erythrocyte/Hela cancer cell	Mesoporous silica gold nanorods	Sonication and extrusion	DOX	Intravenous	Homologous targeting; Long circulation; Chemo-photothermal therapy	Cervical cancer	(51)
RAW 264.7 cell/CT26 cancer cell	MSNs layered persistent luminescence NPs	Extrusion	Photothermal fluorescent dye IR825 and irinotecan	Intravenous	Homologous targeting; Immune evasion; Imaging-guided photothermal therapy	Colorectal cancer	(52)
Bone marrow-derived dendritic cells from the 4T1 cancer cell	MSNs	Extrusion	Immune adjuvant R837	Intravenous	Promoted antigen endocytosis into dendritic cells; Immunotherapy	Breast cancer	(53)
RAW 264.7 cell/platelet	Dendritic MSNs	Sonication	A near infrared fluorescent dye IR780 and DOX	Intravenous	Special targeting of TNBC cells; Crossing vascular barriers; Combination of PTT/PDT and chemotherapy	TNBC	(54)

MSNs, mesoporous silica nanoparticles; PDT, photodynamic therapy; MSNR, mesoporous silica nanorods; DOX, doxorubicin; ICG, indocyanine green; PTT, photothermal therapy; FA, folic acid; GPC3, Glypican-3; HCC, hepatocellular carcinoma; TNBC, triple negative breast cancer.

## 2 Tumor immune microenvironment

### 2.1 Composition of tumor immune microenvironment

Tumor cells are the central players in the TIME. They can secrete a variety of factors that shape the microenvironment to their advantage (55, 56). Tumor cells often overexpress immune checkpoint molecules such as programmed death - ligand 1 (PD - L1), which binds to programmed death - 1 (PD - 1) on T cells, leading to T cells exhaustion and immune evasion (57, 58). Immune cells in TIME include macrophage, neutrophils, T cells, natural killer (NK) cells, B cells, dendritic cells (DCs), and myeloid-derived suppressor cells, which impacts tumor development, invasion, metastasis, and outcome (59, 60). These cells are functionally divided into anti-tumor subsets and pro-tumor immunosuppressive subsets (61). The anti-tumor subsets are characterized by CD8^+^ T cells (62), mature DCs (63), M1-type macrophages and NK cells (64, 65), which drive tumor clearance. Meanwhile, the pro-tumor immunosuppressive subsets are characterized by recruit regulatory T cells (Tregs) (66), M2-type macrophages and myeloid-derived suppressor cells (MDSCs), which promote immune evasion and tumor progression (67). They have broad and complex functional status, which interact with each other and cancer cells through inflammatory factors, chemokines, exosomes and other factors, playing key regulatory roles in tumor inflammation, cancer cell immune evasion, angiogenesis, cancer cell invasion and metastasis (68). Leveraging immune cells’ diversity and versatility, immune cell membrane-coated MSNs exhibit cancer, inflammation, and TIME targeting properties, enabling high specificity in cancer therapy.

### 2.2 Soluble factors

Within the TIME, cytokines precisely orchestrate the recruitment and positioning of immune cells through the establishment of chemokine gradients, a critical step in initiating immune responses (69). Specifically, chemokines such as CCL2, secreted by tumor and stromal cells, bind to their receptor CCR2 to efficiently recruit monocytes to the tumor site. These monocytes subsequently differentiate into immunosuppressive tumor-associated macrophages (TAMs) and help recruit regulatory T cells (Tregs), collectively suppressing antitumor immunity (70). Conversely, IFN-γ produced by activated immune cells induces the generation of chemokines like CXCL9 and CXCL10 by stromal cells (71). These chemokines act as specific ligands for the CXCR3 receptor on Th1 cells and cytotoxic T cells, guiding the infiltration of these effector cells into the tumor core to directly mediate tumor cell killing (72). However, inhibitory cytokines such as TGF-β and IL-10, which coexist in the TIME, not only directly suppress T cell function but also disrupt the normal expression of these beneficial chemokines (73). This disruption impairs productive immune cell recruitment, ultimately leading to insufficient effector T cell infiltration and immune tolerance. Thus, the balance of the chemokine network directly dictates the immune cell composition within the TIME and the ultimate outcome of the antitumor immune response (74).

In addition, vascular endothelial growth factor (VEGF), a key mediator of tumor angiogenesis, also critically promotes Treg generation (75). VEGF binds to its receptors on naive T cells and bone marrow-derived precursor cells, activating downstream signaling pathways that drive their differentiation into functional Tregs (76). These VEGF-induced Tregs further accumulate in the TIME, where they collaborate with TAMs and inhibitory cytokines to reinforce the immunosuppressive network by suppressing effector T cell activation, proliferation and cytotoxic function while impairing the production of beneficial chemokines such as CXCL9 and CXCL10 (77, 78). Collectively, the interplay between chemokines, inhibitory cytokines and VEGF underscores the complexity of the TIME microenvironment, where the dysregulation of multiple signaling axes converges to hinder effective antitumor immune responses and promote immune tolerance (73).

### 2.3 Immune escape mechanisms in TIME

Tumors employ multiple immune escape mechanisms in TIME (79, 80). One of the key mechanisms is the upregulation of immune checkpoint molecules, targeting specific immune checkpoint receptors has become the main method to regulate immune system function in cancer treatment (81). Another critical mechanism is the downregulation of major histocompatibility complex (MHC) molecules on tumor cells, which impairs the presentation of tumor-specific antigens to cytotoxic T cells and Th1 cells, making tumor cells undetectable by the adaptive immune system (82, 83).

Furthermore, tumors widely express “don’t eat me” signals such as CD47, which binds to signal regulatory protein α (SIRPα) on macrophages to inhibit phagocytic activity, enabling tumor cells to evade clearance by the innate immune system (84). Tumors also actively recruit immunosuppressive immune cells such as tumor-associated macrophages (TAMs) (85), myeloid-derived suppressor cells (MDSCs) (86), and regulatory T cells (Tregs) into the TIME (87), where these cells further suppress antitumor immunity through direct cell-cell interactions or the secretion of inhibitory factors (88). And hypoxia further induces tumor cells and stromal cells to upregulate inhibitory molecules like PD-L1, enhances the recruitment and polarization of immunosuppressive cells including TAMs and MDSCs, and directly impairs the metabolic activity and cytotoxic function of effector T cells (89).

Additionally, the immunosuppressive cytokines and chemokines in the tumor immune microenvironment can inhibit the recruitment, activation, and function of immune cells (90, 91). The physical barriers formed by the extracellular matrix and stromal cells can also prevent immune cells from accessing tumor cells (92, 93). Apoptosis or functional failure of immune cells induced by tumor cells will also further promote immune escape (94).

## 3 Interface interaction between cell membrane-biomimetically modified MSNs and biological membranes and its immunological significance

### 3.1 Immune recognition and response of biological membranes to foreign nanoparticles

In unmodified state, bare mesoporous silica nanoparticles are recognized as foreign entities. Their interactions with biological membranes are primarily driven by non-specific physicochemical forces, such as electrostatic interactions and van der Waals forces (95, 96). This interaction typically triggers a series of immune responses, the core of which is the formation of a protein corona (97). When MSNs enter biological fluids, their surfaces rapidly adsorb a layer of biomolecules—such as immunoglobulins (98), complement proteins (99), and fibrinogen (100)—forming the so-called “protein corona”. This corona fundamentally alters the original identity of the nanoparticles, enabling their recognition by immune cell surface receptors, including Fc receptors and complement receptors. This leads to clearance via endocytosis and may elicit a pro-inflammatory response (101).

Cell membrane coating fundamentally alters this process. By providing a native lipid bilayer and membrane proteins bearing self-markers, it effectively shields the foreign surface of MSNs and minimizes the adsorption of non-specific proteins (102). For instance, MSNs coated with erythrocyte membrane extracts, as demonstrated by Zhang et al. (103), retain key “don’t eat me” signal proteins such as CD47 on their surface. The interaction between CD47 and signal regulatory protein α (SIRPα) on macrophages directly transmits an inhibitory signal, significantly reducing phagocytosis of the nanoparticles by macrophages and prolonging their systemic circulation time. Consequently, the primary immune response of biological membranes towards cell membrane-coated MSNs shifts from an active “clearance” mode to a more “tolerogenic” mode, thereby enhancing their ability to effectively target the tumor immune interface (104).

### 3.2 Key glycoprotein in immune interface constructed by cell membrane coating

The immunomodulatory capabilities demonstrated by cell membrane-coated MSNs originate from the parental cell membrane proteome, particularly glycoproteins, inherited on their surfaces (105). These glycoproteins constitute the “molecular language” enabling specific interactions between the nanoparticles and immune cell membranes (106). CD47 and CD200 are primary glycoproteins mediating immune evasion. In various membrane coating strategies (84, 107), CD47 derived from erythrocyte membranes or certain cancer cell membranes has been proven critical for endowing nanoparticles with long circulation capabilities (108). CD200, another significant immunomodulatory molecule widely expressed on neurons, endothelial cells, and certain immune cells, binds to the CD200 receptor on myeloid cells (109). This interaction inhibits macrophage activation and the production of inflammatory factors. Coating with CD200-expressing cell membranes can actively suppress the pro-tumoral (M2) phenotypic polarization of tumor-associated macrophages (110).

Furthermore, the integrin family and selectin ligands primarily mediate targeted adhesion and signal transduction. When leukocyte membranes or mesenchymal stem cell membranes are used for coating, MSNs retain integrins such as LFA-1 and VLA-4 on their surfaces (111, 112). These proteins recognize adhesion molecules (e.g., ICAM-1, VCAM-1) on immune cells or endothelial cells. This not only confers upon the nanoparticles the ability to actively target inflammatory or tumor sites but also enables the binding process itself to transmit intracellular signals, influencing the migration and activation status of immune cells (113). Leukocyte membranes are enriched with glycoproteins like PSGL-1, which serve as high-affinity ligands for P-selectin and E-selectin (114). Given the frequent overexpression of E-selectin on tumor vascular endothelial cells, membrane-coated MSNs carrying selectin ligands can mimic the leukocyte “rolling” behavior (115). This facilitates their initial retention at tumor vasculature, establishing a foundation for subsequent transendothelial transport (116).

In summary, cell membrane coating transforms MSNs into biomimetic platforms that enable both immune evasion and active targeting. Through rational design of surface glycoproteins and particle characteristics, these systems achieve targeted delivery to the tumor microenvironment and mediate precise immunomodulation via native ligand-receptor interactions.

### 3.3 The role of membrane-coated MSNs in immune regulation

MSNs exert multifaceted and tunable regulatory effects on both innate and adaptive immunity (117). MSNs hold inherent adjuvant properties, and directly regulate the functional state of key immune cell subsets including macrophages and dendritic cells (118, 119). They effectively promote antigen presenting cell maturation, enhance antigen uptake and presentation efficiency, drive robust Th1 type cellular immune responses, and promote the clonal expansion of antigen specific T cells to strengthen long term immune memory formation (120, 121). MSNs precisely modulate proinflammatory and anti-inflammatory cytokine secretion to remodel the immune microenvironment, and mediate bidirectional immune regulation via orchestrating crosstalk between innate and adaptive immunity (122, 123).

Membrane coated MSNs present distinct advantages over conventional nanoparticles including liposomes and polymeric nanoparticles in immune related applications. Liposomes often suffer from rapid cargo leakage and poor structural stability in systemic circulation, while polymeric nanoparticles face challenges in inter-batch reproducibility and low drug encapsulation efficiency (124, 125). Unlike these conventional delivery systems, the uniformly ordered and tunable porous structure of MSNs enables high efficiency co delivery of diverse cargoes, supporting versatile multimodal therapeutic regimens especially for cancer immunotherapy (126). The rigid framework of MSNs maintains structural integrity under harsh physiological conditions, ensuring stable cargo release at target sites (127). They also possess flexible surface functionalization and intrinsic adjuvant activity, which reduce systemic toxicity (128). Membrane coating further endows MSNs with prolonged circulation time and reduced off target clearance (129). These merits collectively establish membrane-coated MSNs as a more advantageous delivery platform.

## 4 Strategies of membrane-coated MSNs for targeting tumor immune microenvironment

**Figure 2:**
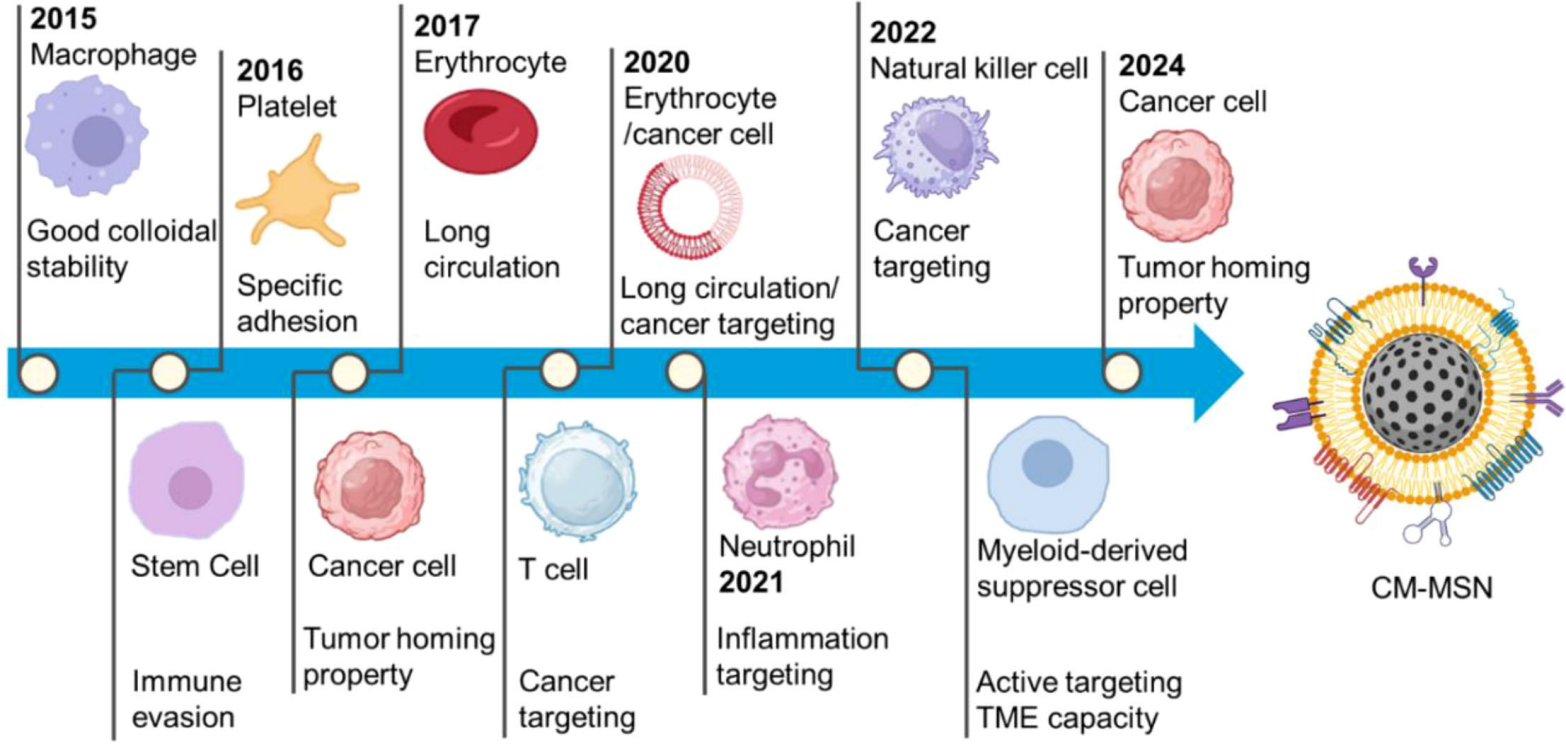
Different cell membrane firstly utilized in CM-MSNs fabrication. These cell membranes derived from cells like macrophage (130), stem cell (131), platelet (38, 132), erythrocyte (133), cancer cell (134–137), T cell (44), erythrocyte/cancer cell (51), neutrophil (43), myeloid-derived suppressor cell (138) and natural killer cell (45) have been used as coating materials of MSNs for cancer therapy (image was created with BioRender.com).

The macrophage membrane coated MSNs which loaded with doxorubicin (DOX) was firstly designed for breast cancer therapy in 2015 (130). Subsequently, the timeline of publications documents the exploration of cell membranes derived from stem cells, platelets, erythrocytes, cancer cells, T cells, neutrophils, and natural killer cells as coating materials for MSNs in cancer therapy (**Figure 2**). Cell membrane coating can endow CM-MSN with colloidal stability, long circulation time, immune evasion, cancer targeting and inflammation targeting functions. Artificially synthesized MSNs can be loaded with various chemotherapy drugs, photothermal agents, photosensitizers, antibodies, or alter physicochemical properties to exert chemotherapy, photothermal, and other anti-tumor effects.

### 4.1 Preparation methods of membrane-coated MSNs

Common preparation strategies for membrane-coated mesoporous silica nanoparticles mainly include extrusion, sonication, and incubation methods. Extrusion achieves uniform membrane coating via repeated mechanical extrusion of membrane-nanoparticle mixtures through polycarbonate porous membranes (139). Sonication completes membrane wrapping through ultrasonic driven fusion between extracted membrane vesicles and mesoporous silica nanoparticle surfaces (140). Incubation forms a biomimetic coating via mild physical adsorption under gentle stirring without additional energy input (141). Extrusion methods provide uniform coating thickness, intact membrane structure and excellent inter-batch reproducibility, while preserving mesoporous channel integrity and drug loading capacity (142).

### 4.2 Targeting mechanism and polarization regulation of macrophage coating

TAMs are the main type of tumor-infiltrating immune cells, generally categorized into M1 and M2 phenotypes. M1-type macrophages typically exert tumor-suppressive effects and pro-inflammatory functions, while M2-type macrophages drive tumor progression and exert anti-inflammatory effects (143). Macrophage colony-stimulating factor 1 (CSF1), which is secreted by cancer cells, interacts with CSF1 receptor (CSF1R) expressed on the macrophage membrane and further maintains the immunosuppressive function of TAMs (144). Inspired by this interaction of CSF1 with CSF1R, macrophage membranes (MM) have been used to modify various nanoparticles for cancer targeting and regulating the polarization of TAM towards the M1 phenotype which can exert the tumor antagonistic effect (145). For example, the M1 macrophage membrane (M1-MM) was obtained from RAW 264.7 cells induced by lipopolysaccharide, which coated on the surface of nanoparticles showed 1.87 folds uptake by M2 macrophage compared with bare nanoparticles. This result indicated that nanoparticles enveloped with M1-MM could be specifically taken up by TAMs, which is beneficial for targeted drug delivery (146). Yoon et al. enhanced the TAM targeting ability of MM by modifying MM with mannose (a ligand can selectively target TAM), and further coated it on the surface of silica layered upconverting nanoparticles which co-doped with perfluorocarbon (PFC)/Ce6 and loaded with paclitaxel (G5-UCNPs@mSiO2-PFC/Ce6@RAW-Man/PTX) for PDT. Compared with bare nanoparticles, UCNPs@mSiO2-PFC/Ce6@RAW-Man/PTX exhibited significantly increased cellular uptake by M2 macrophages due to the mannosylated MM coating (40).

**Figure 3:**
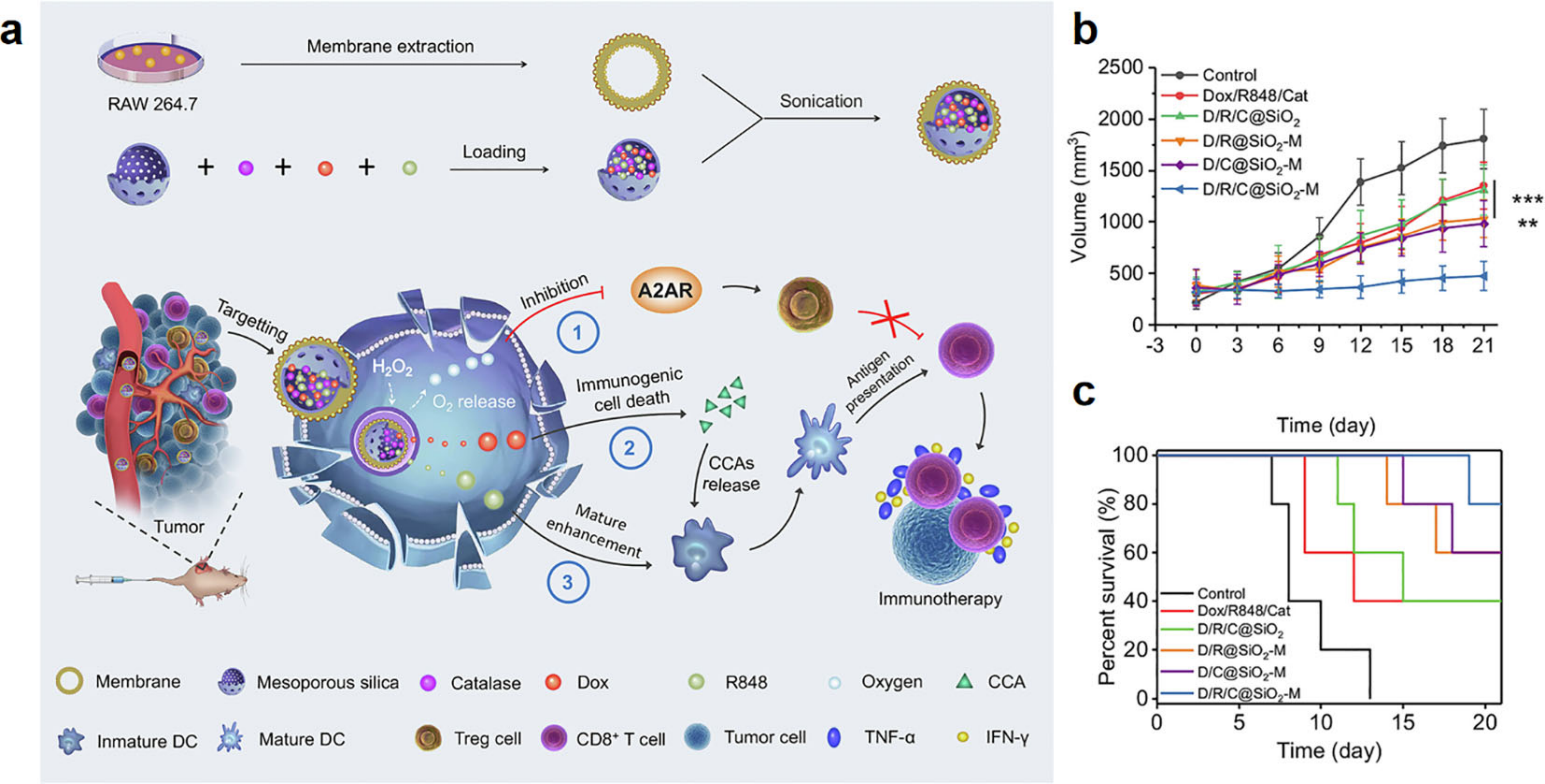
Macrophage membrane coated MSNs for cancer therapy. **(a)** Schematic illustration of the multifunctional nanoplatform preparation and its principle of enhancing immunotherapy efficacy (41). **(b)** Tumor volume in different treatment groups (**p < 0.01 and ***p < 0.001 vs. the D/R/C@SiO2-M group) (n = 5) (41). **(c)** Survival curves of the mice treated with different formulations (n = 5) (41). ^©^ 2022 Wen et al. Published by Elsevier B.V.

MM shows high expression of α4 and α4β1 integrin that can specifically bind to VCAM-1 on cancer cells (147). Wen et al. constructed a drug delivery system based on MM coated MSNs (D/R/C@SiO2-M) (41), the MM coating not only reduced the drug leakage but also enhanced cancer targeting of D/R/C@SiO2-M (**Figure 3a**). The inhibitory rate of D/R/C@SiO2-M on tumor growth reached 73.58%, which was 2.68 folds higher than that of bare MSNs, indicating that the cancer-targeting capability of MM contributed to the inhibition of tumor growth (**Figures 3b, c**).

Previous studies have shown that the rod-shaped MSNs (MSNRs) have better tumor penetration ability and higher cell uptake than spherical MSNs (148, 149). Considering the specific performance of MSNR, Li et al. prepared a nanoplatform based on MSNR with MM coating for thermos-chemotherapy of breast cancer *in vivo*. The nanoplatform integrated high cell uptake and tissue specific targeted accumulation, exerting enhanced effect of thermos-chemotherapy (42). In addition to macrophage cell lines cultured *in vitro*, some primary macrophages from *in vivo* sources, such as primary TAMs or bone marrow-derived macrophages (BMDMs) can also exert unique cancer targeting ability through VCAM-1/α4β1 integrin interaction (150). And it is necessary for researchers to carefully consider the source of cell membranes because the heterogeneity of white blood cell functions is determined by the source of cells (151).

### 4.3 Inflammatory targeting and blood-brain barrier penetration of neutrophil coating

Neutrophils, as one of the most common immune cells, play an important role in inflammation. Neutrophils interact with ICAM-1 and ICAM-2 expressed on endothelial cells through integrin LFA-1, tightly adhering to vascular endothelium, and transferring from blood circulation to inflammatory tissue (152). TME is characterized by chronic inflammation, neutrophils recruit to the TME by responding to various proinflammatory chemokines and cytokines (153). Thus, more and more studies have taken advantage of the inflammatory targeting capability of neutrophil cell membranes (Nm) to develop novel drug delivery system for cancer therapy (154). Jiang et al. designed a novel drug delivery system composed of DOX and anti-inflammatory drug Shanzhiside methylester co-loaded MSNs with Nm coating (Nm@MSNs-DOX/SM) for chemotherapy and anti-inflammatory therapy in SU-DHL-2-tumor-bearing mice. The apoptosis rate of SU-DHL-2 cells treated by Nm@MSNs-DOX/SM group and MSNs@DOX/SM group was 46.7 ± 3.2% and 30.5 ± 2.6% *in vitro*, respectively. Anti-tumor effect was enhanced due to inflammatory targeting capability and reduced macrophage phagocytosis by Nm modification (43).

In addition to inflammation targeting, neutrophils can also cross physiological barriers such as blood-brain barrier (BBB) and blood-tumor barrier (BTB) to infiltrate solid tumors. Nm coated nanoparticles inherited the BBB/BTB penetrating ability of neutrophils, which could be used for the brain glioblastoma treatment (155, 156). In conclusion, Nm is potential candidate in CM-MSNs based drug delivery system against cancer due to their high mobility towards inflammatory sites and ability to trespass the BBB and BTB. Unlike drug delivery systems based on living neutrophils, which may be reprogrammed as immunosuppressive phenotypes after metastasis to TME, posing additional risks to cancer patients, Nm coated MSNs are safer and more effective (157, 158).

### 4.4 Targeting and time accumulation of T cells and NK cell membrane coatings

**Figure 4:**
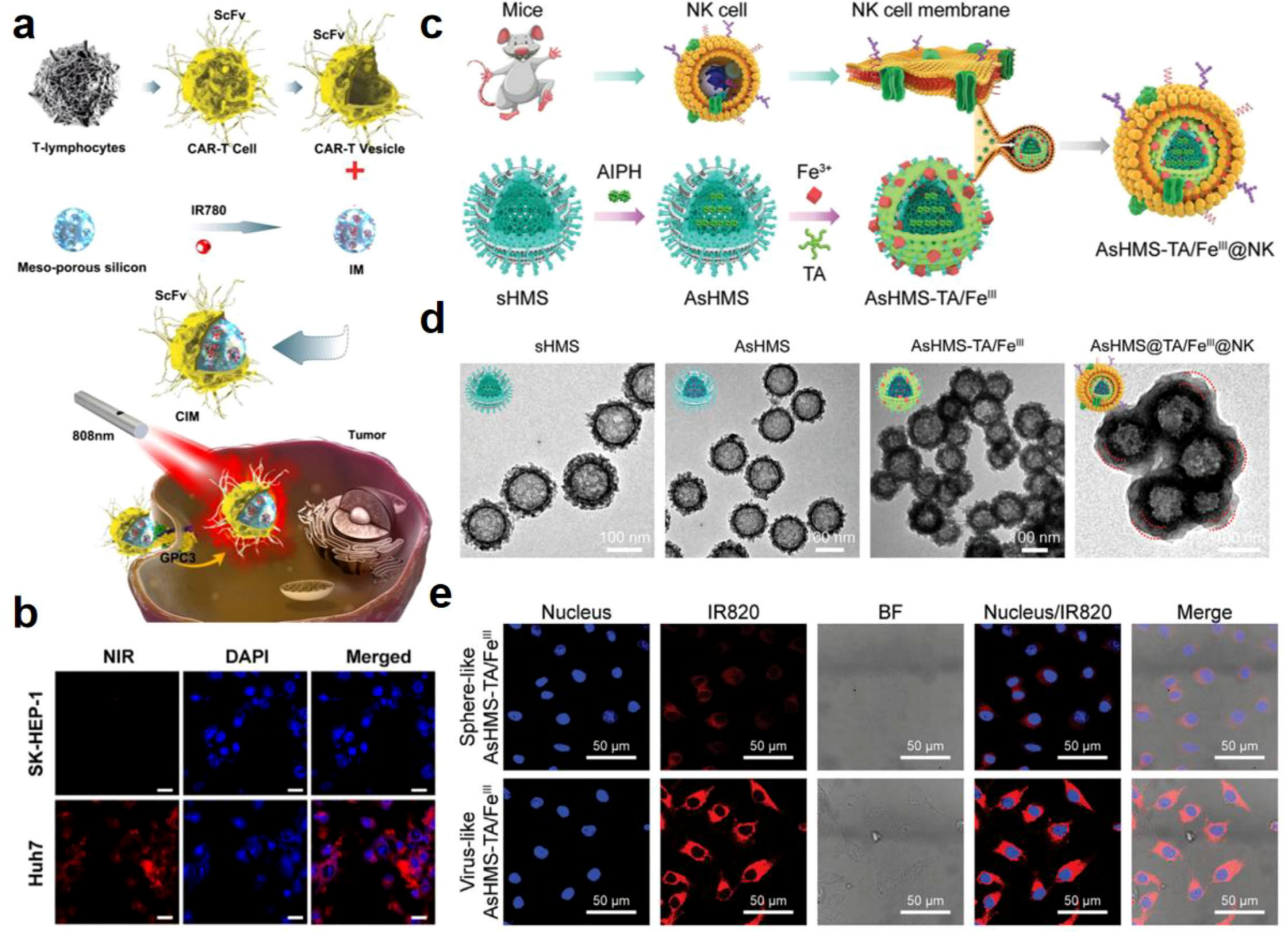
CAR-T cells and NK cell membrane coated MSNs for cancer therapy. **(a)** Schematic illustration of chimeric antigen receptor-T (CAR-T) membrane coated biomimetic nanoparticles for highly specific tumor photothermal therapy (44). **(b)** CLSM images of Huh-7 cancer cells and SK-HEP-1 cells after incubation with GPC3-CAR-T membrane-coated IMs (CIMs). Scale bar: 20 μm (44).^©^ 2020 Ma et al. Theranostics published by Ivyspring International Publisher. **(c)** Schematic illustration of the synthesis of NK cell membrane-cloaked virus-mimicking AsHMS-TA/FeIII@NK nanogenerator (45). **(d)** TEM images of sHMS and AsHMS-TA/FeIII@NK (45). **(e)** CLSM images of HepG2 cells incubated with sphere-like and virus-like nanogenerators for 4 h. Reproduced with permission (45). ^©^ 2021 Lin et al. Advanced Science published by Wiley-VCH GmbH.

Chimeric antigen receptors (CARs) provide defined antigenic specificities for T cell populations to target tumors, CAR-T cells can specifically recognize tumor related antigens and eliminate tumor cells through single chain variable region (ScFv) (159). Based on these mechanisms, T cell reprogramming strategy against cancers including T cell derived membrane-based drug delivery system have been developed recently (160). Glypican-3 (GPC3) is expressed in 75% of hepatocellular carcinoma cells, but not in normal tissues (161). To selectively bind to GPC3-positive HCC cells, Ma et al. first isolated primary human T cells from peripheral blood mononuclear cells (PBMCs) of healthy donors via negative selection, then activated the primary T cells by anti-CD3/anti-CD28 antibodies, and subsequently transduced them with GPC3-CAR lentivirus polybrene to construct functional GPC3-targeted CAR-T cells. Subsequently, a novel drug delivery system for precise *in vitro* and *in vivo* tumor imaging and targeted therapy was constructed, with a biodegradable core of high drug-loading IR780-loaded mesoporous silica nanoparticles (IMs) and a GPC3-CAR-T cell membrane shell coated via extrusion (**Figure 4a**). Results showed that the *in vitro* tumor targeting ability of GPC3-CAR-T membrane-coated IMs (CIMs) was 10 times that of uncoated IMs; under NIR laser irradiation, CIMs exhibited a 33% higher tumor inhibition rate than IMs (**Figure 4b**) (44).

NK cells are innate lymphocytes with the ability to recognize tumor cells and accumulate into the tumor tissues under the guidance of chemokines such as chemokine (C-X-C motif) ligand 9 and chemokine (C-X-C motif) ligand 10 secreted by DCs (88). Clinical research is currently underway on the treatment of cancer with adoptive transfer of autologous or allogeneic NK cells (162). Inspired by these cases, Lin et al. reported a multilevel intelligent responsive nanoarchitectures (AsHMS-TA/FeIII@NK) consisted of NK cell membrane coating and tannic acid (TA)/Fe^3+^ photothermal assembly decorated virus-like disulfide-doped hollow MSN with azoinitiator (AIPH, a radical precursor) loaded inside (**Figure 4c**) (45). A virus-like surface morphology of MSN could be observed, further transformed into a nearly spherical morphology after NK cell membrane coating (**Figure 4d**). The results showed that the tumor accumulation of AsHMS-TA/FeIII@NK was significantly higher than that of AsHMS-TA/FeIII, as the NK cell membrane coating enabled the nanosystem to effectively evade macrophage phagocytosis and reduce the secretion of pro-inflammatory cytokines in macrophages, thus alleviating immune responses and facilitating its targeted delivery and retention in TME (**Figure 4e**).

### 4.5 Tumor-specific targeted adhesion and immune evasion mediated by platelet membrane coated

Platelet can selectively adhere to tumor tissues and damaged vasculatures through receptors such as glycoprotein Ib (GPIb), glycoprotein Ia/IIa (GPIa/IIa), CD41, and CD61 (163). Li et al. loaded vascular disruption agents (VDAs) and anti-angiogenic drug (AAD) into platelet membrane (PM) coated MSNs (MSN@PM-C-A) for liver tumor elimination. With the help of PM coating, MSN@PM-C-A adhered to the tumor tissues. Subsequently, VDA and AAD destroyed blood vessels and prevent angiogenesis, leaving the tumor blood vessels in a state of destruction. MSN@PM-C-A was continuously recruited by damaged tumor blood vessels due to the specific adhesion of PM coating, resulting in a high accumulation of drug for effective tumor eradication (35).

**Figure 5:**
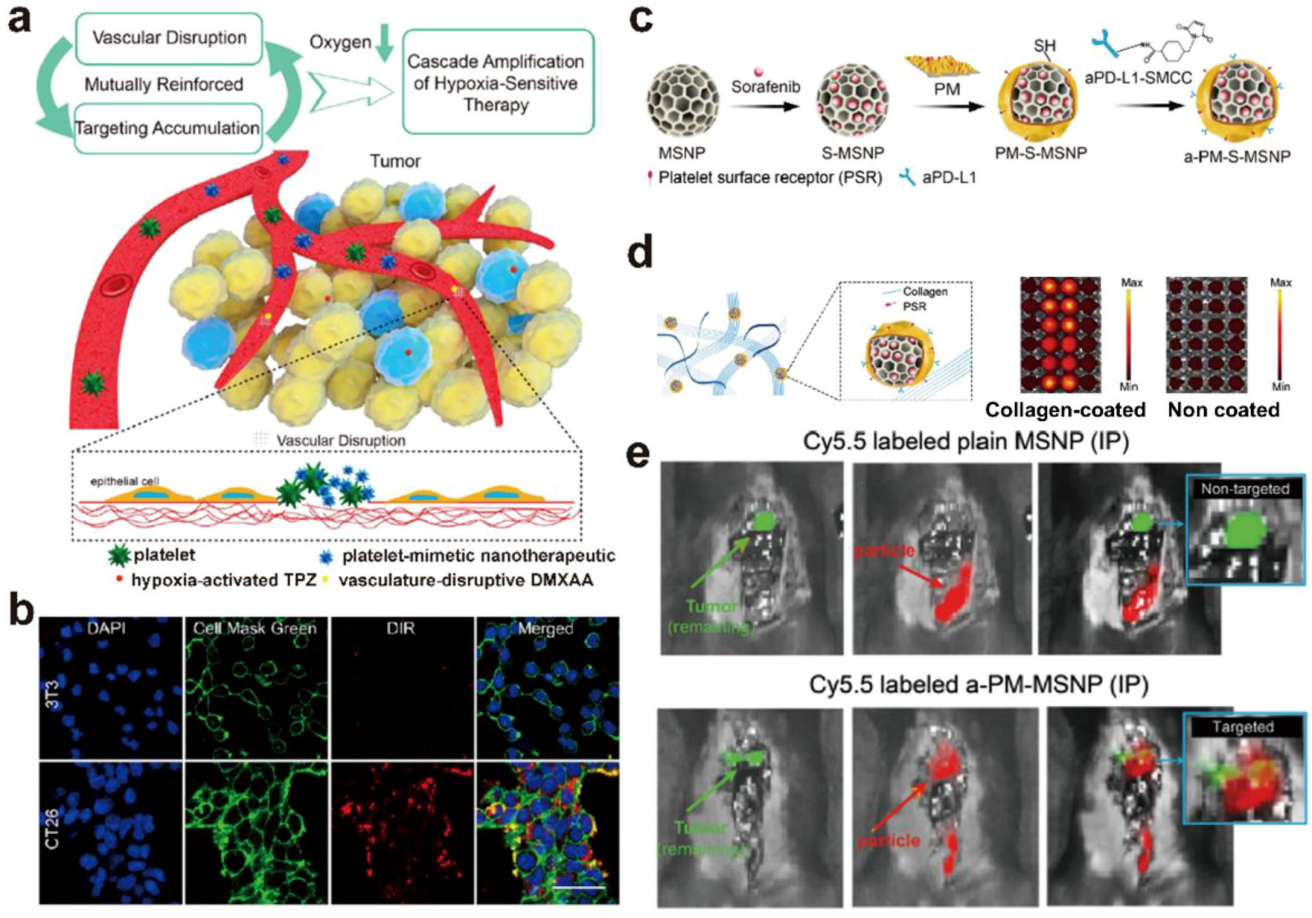
Platelet membrane coated MSNs for cancer therapy. **(a)** Illustration of bioinspired nanodesign for biologically driven cooperation among antitumor vascular disruption, platelet-like biotaxis, cascade hypoxia amplification, and hypoxia-sensitive chemotherapy (36). **(b)** Fluorescence images of 3T3 (upper) and CT26 (lower) cells after incubation with 100 μg/mL of MTD@P for 2 h (36). ^©^ 2019 Zhang et al. ACS Nano published by American Chemical Society. **(c)** Schematic illustration of the synthesis process of a-PM-S-MSNP nanodrug (37). **(d)** Schematic of binding between a-PM-S-MSNP and collagen. Platelet membrane coated on particle surfaces can bind to the exposed collagen of surgical margin largely through the platelet surface receptor (PSR) such as GPIb, GPIa-IIa, CD41, and CD61. And representative imaging data of *in vitro* binding in collagen coated (left) versus non-coated plates (right) for a-PM-S-MSNP and various controls. Our data revealed a strong binding affinity between a-PM-S-MSNP and collagen IV coated plates (bright red) (37). **(e)** Targeting effect of Cy5.5 labeled plain MSNP or a-PM-MSNP after IP injection at 30 mg kg−1 in surgical removal model. Eight hours post-injection, the signals of nanoparticles (red) and tumor tissues (green) were obtained. a-PM-MSNP gave obvious tumor lesion targeting compared with the control (37). ^©^ 2022 Li et al. Advanced Science published by Wiley-VCH GmbH.

Tumor blood vessels rupture is accompanied by platelet recruitment and the resulting inhibition of oxygen supply. Inspired by this process, Zhang et al. constructed MTD@P by co-loading amino-modified mesoporous silica nanoparticles with tirapazamine and vascular-disrupting agents, then coating the nanoparticles with extracted mouse platelet membrane via sonication and extrusion (36). The cellular uptake of MTD@P in CT26 cancer cells was 15 folds higher than that in normal 3T3 cells *in vitro* as the interaction of P-selectin on PM with CD44 overexpressed on cancer cell membrane, indicating a cancer targeting characteristic of MTD@P. MTD@P was recruited into tumor blood vessels through the interaction of glycoprotein VI with exposed extracellular matrix, and further destroyed the tumor blood vessels, resulting in tirapazamine release and 20 folds higher tumor hypoxia effect than that resulted by bare MSNs. A cascade amplification of hypoxia-sensitive therapy combined with enhanced chemotherapy was realized by such a dual targeting system (**Figures 5a, b**).

In addition to damaged tumor blood vessels, platelet also actively target to postoperative wounds based on the interaction of CD41, CD61, and CD62p with type IV collagen exposed after surgery. Inspired by these interactions, Li et al. designed a system (a-PM-S-MSNP) for the treatment of postoperative recurrence of cancer. Briefly, they synthesized sorafenib-loaded MSNPs, coated the nanoparticles with platelet membranes at an optimal mass ratio via ice-bath sonication, and further conjugated aPD-L1 antibodies to construct the final a-PM-S-MSNP. The co-localization of a-PM-S-MSNP and tumor, as well as non-co-localization of bare MSNP and tumor in a postsurgical HCC mouse model, indicated the specific accumulation of PM coated MSNs in postoperative wounds *in vivo* (**Figures 5c–e**) (37). PM coatings protect nanoparticles from rapid blood clearance due to the presence of CD47 on PM and interact with cancer cells by molecular interactions, such as the specific binding of CD62p receptors from platelet with CD44 receptors that overexpressed on the surface of cancer cells (19, 164). Chen et al. constructed a multifunctional platform consisted of bismuth sulfide nanorods which modified with PM coated mesoporous silica layer. The content of BMSNR@PM was 2.1 folds higher than that of bare nanoparticle in tumor sites, indicating a superior immune evasion and cancer targeting ability of PM coating (38).

Furthermore, platelets can adhere to circulating tumor cells (CTCs) and promote tumor metastasis (165). Inspired by the interaction of platelets and CTCs, Da et al. functionalized PM with aPD-L1 (a-PM) though a maleimide linker, the a-PM was further used as the coating material of MSNs for achieving functions of CTCs targeting and immune evasion. To evaluate the targeting ability of PM-coated MSNs, they analyzed lung images from mice injected with H22 cancer cells. Results showed that PM coating significantly increased the co-localization area of MSNs in fibrin by 619.34 mm^2^, indicating effective CTCs targeting of MSNs by PM coating (39). In summary, PM coated MSNs provide a versatile drug delivery system for cancer cell-targeted treatment through specific adhesion of PM coating to damaged tumor blood vessels and CTCs.

### 4.6 Circulating prolongation and tumor-targeted accumulation ability of cancer cells membrane coating

**Figure 6:**
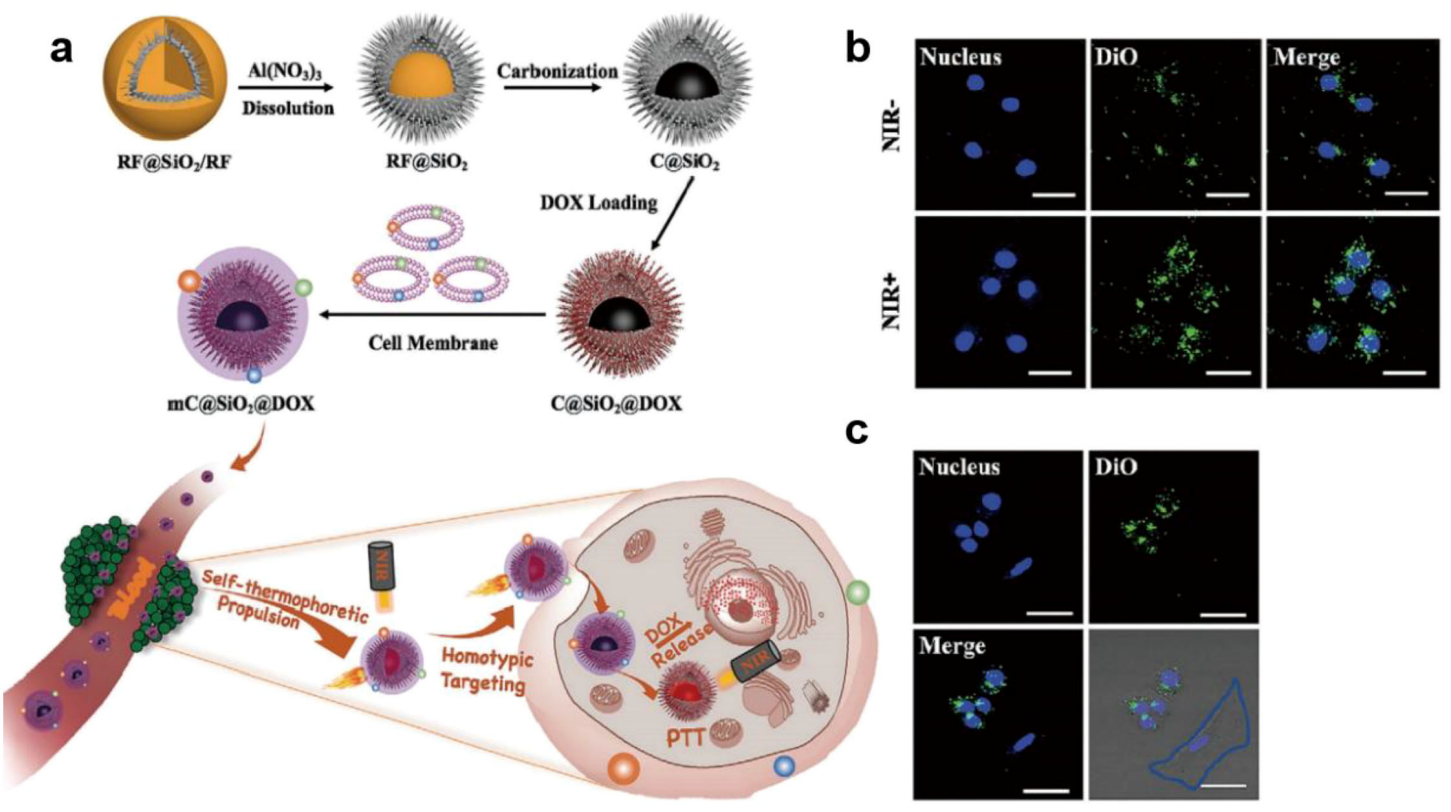
Cancer cell membrane coated MSNs for cancer therapy. **(a)** Schematic illustration of the mC@SiO2@DOX nanomotors preparation and its principle of enhancing photo - chemotherapy immunotherapy efficacy (47). **(b)** CLSM images of the prestained mC@SiO2@DOX (50 µg/mL) adhered to the surface of MCF-7 cells with or without NIR laser irradiation (980 nm, 0.8 W/cm−2, 30 min). Scale bar: 50 µm (mC@SiO2@DOX dyed with DiO and nucleus dyed with Hoechst 33342) (47). **(c)** CLSM images of MCF-7 and NHDF cells after incubated with mC@SiO2@DOX during NIR laser irradiation (980 nm, 0.8 W/cm−2, 30 min) and then cultured for another 3.5 h. The NHDF cell is surrounded by a blue box. Scale bar: 50 µm. Reproduced with permission (47). ^©^ 2020 Zhou et al. Small published by Wiley-VCH GmbH.

Cancer cells express various functional proteins, such as N-cadherin, galectin-3, and epithelial cell adhesion molecule (EpCAM), which mediate homologous binding, and CD47, a biomarker of self-recognition and immune evasion (166). In addition, it is easy to isolate and obtain enough cell membranes because of the infinite proliferation and rapid expansion of cancer cells *in vitro (*167, 168). Therefore, there have been a large of studies applied cancer cells membrane (CCM) coated nanoparticles to cancer therapy (169). For example, Liu et al. described a tumor acidic environment responsive drug delivery system (DOX/MSN@CaCO3) that CaCO3 layered MSNs loaded with DOX and then coated with prostate cancer cells (LNCaP-AI cell line) membrane. The CCM could prevent drug leakage under normal physiological conditions, and guide DOX/MSN@CaCO3 specifically targeting tumor sites (46). The drug leakage inhibition ability and tumor homing property of CCM effectively reduce the toxic side effects of chemotherapy, such as DOX, which can cause cardiac toxicity, inducing cardiomyopathy and even leading to congestive heart failure (170). Moreover, considering the tumor homing property of CCM, Zhou et al. designed a NIR light induced self-propulsion nanomotor based on MCF-7 cancer cell membrane coated carbon silica with DOX loading (mC@SiO2@DOX) (**Figure 6a**) (47). In this system, MCF-7 cancer cell membrane coating exhibited specific self-recognition of MCF-7 cancer cells. The core carbon silica with asymmetric structure achieved NIR light induced self-propulsion. Under NIR light, the self-propulsion ability of core nanoparticles increased the uptake efficiency of MCF-7 cells to mC@SiO2@DOX *in vitro* from 26.2% to 67.5% compared to no self-propulsion ones (**Figure 6b**). Compared with normal human dermal fibroblasts, mC@SiO2@DOX was preferential to accumulate in the cytosol of MCF-7 breast cancer cells (**Figure 6c**), indicating the self-recognition property of cancer cell membrane coating integrity.

Usually, CM-MSN can be used for immunotherapy though immune adjuvants loading and specific antibodies modification. For example, Xie et al. supported that B16-F10 CCM coated MSNs could delivery glucose oxidase to enhance the therapeutic effect of PD-1 immunotherapy on B16-F10 tumor bearing mice (48). Also, Cheng et al. designed a novel drug delivery system (DLMSN@CuS/R848) integrating photothermal ablation and immune remodeling for the treatment of metastatic triple negative breast cancer (TNBC). In this system, dendritic MSN (DLMSN) loaded with copper sulfide (CuS) nanoparticles and immune adjuvant rekimod (R848), and 4T1 CCM conjugated with anti-PD-1 peptide AUNP-12 was used as coating material of DLMSN. AM@DLMSN@CuS/R848 reached primary tumor sites under the guidance of 4T1 CCM coating, and CuS nanoparticles exerted photothermal ablation effect under laser irradiation on primary tumor, then the photothermal effect enhanced the fluidity and permeability of the CCM coating and further accelerated the release of R848 to promote the maturation and antigen-presenting functions of DCs, finally inducing immunogenic cell death. Compared with bare DLMSN@CuS/R848, AM@DLMSN@CuS/R848 had a 30% increase in tumor inhibition rate under laser irradiation (49). Similarly, a system that utilizes patients’ own tumor cells as raw materials to develop CMM-MSNs can be applied in clinical cancer therapy, thereby enabling a more effective personalized healthcare approach.

### 4.7 Multiple biological characteristics and functional integration effect of erythrocyte membrane coated

**Figure 7:**
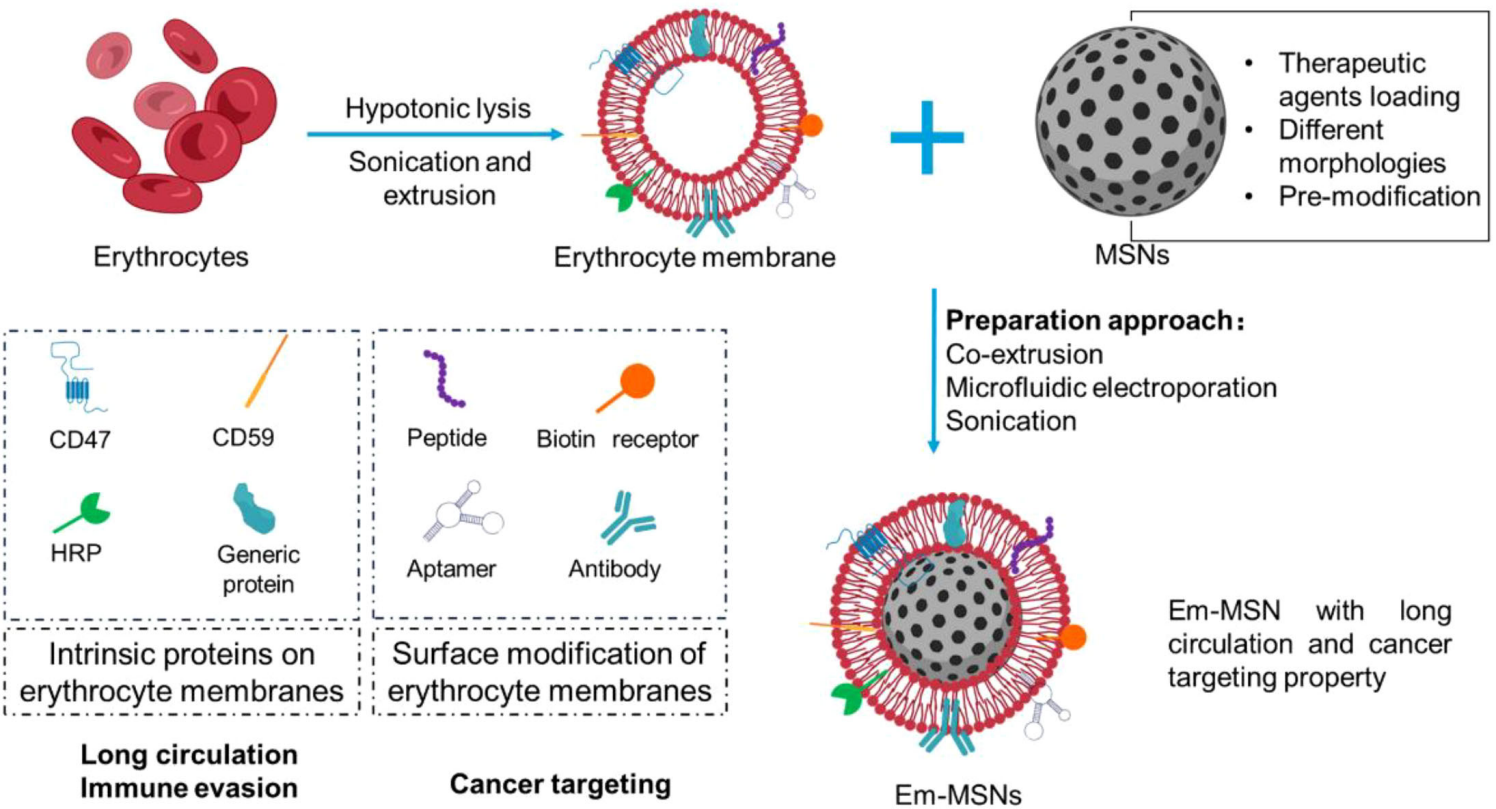
Erythrocyte membrane coated MSNs (Em-MSNs) possess immune evasion and long circulation properties due to the intrinsic membrane proteins such as CD47, CD59 and homologous restriction protein (HRP). Moreover, modification of Em with targeting ligands such as peptide, receptor, and aptamer provide Em-MSNs with cancer targeting ability.

As the most abundant circulating cells in the blood, erythrocytes hold a lifespan of 100–120 days (171). A “don’t eat me” marker CD47 protein is expressed on the surface of erythrocytes, which interacts with the SIRPα receptor, thereby evading phagocytosis of erythrocyte by host cell (172). Moreover, mature erythrocytes lack the contents such as nucleus and endoplasmic reticulum, are easy to obtain relatively well-preserved erythrocyte membrane (Em) structure after hypotonic lysis, and the protein on membrane, such as CD47, is relatively evenly distributed on the surface of nanoparticles after physical co-extrusion (173, 174). Intrinsic proteins like CD47 provide erythrocyte membrane coated MSNs (Em-MSNs) with long circulation property, and targeting ligands modification of Em provide Em-MSNs with cancer targeting ability (**Figure 7**).

Long circulation time is particularly important for cancer treatments, while the mononuclear phagocyte system will quickly clear the injected MSNs, thereby limiting their circulation time and drug delivery efficiency (175). Em-MSNs mimic the erythrocytes and show long circulation property, thus enhancing the efficacy of cancer therapy. For example, non-invasive therapies such as photodynamic therapy (PDT) and photothermal therapy (PTT) have attracted widespread attention from researchers in the treatment of cancers. However, most of photosensitizers and photothermal agents face challenges in immune recognition and rapid clearance in the blood circulation, causing inefficient accumulation of them within tumor sites (176). Study showed that mesoporous silica nanorods (MSNR) loaded with photothermal agent indocyanine green (ICG) and chemotherapeutic drug DOX camouflaged by Em could effectively kill SKBR3 breast cancer cells *in vitro*. The enhanced anti-tumor effect was attributed to the coating of Em, which reduced ICG leakage and macrophage phagocytosis (29). Although it was reported that compared to Em ghosts, the density of CD47 on the erythrocyte membrane decreased by about 30% after co-extrusion with MSNs due to mechanical compression, Em-MSNs still exhibited long-term behavior *in vivo*. The retention of Em-MSNs and bare MSNs in the blood was 1.96% and 1.25% over a span of 54 h, respectively (30). To achieve controlled drug release after the accumulation of Em-MSNs in the tumor sites, the surface of mesoporous silica shell layered upconversion nanoparticles could be pre-covalently grafted with chlorin e6 (Ce6) molecules before Em coating (UCNPs@mSiO2-Ce6). Ce6 generated 1O2 to destroy the Em coating under 980 nm laser irradiation, leading to drug release in the tumor sites. Compared with control group, long circulation time and controlled drug release properties of UCNPs@mSiO2-Ce6 enhanced its effectiveness, with a tumor growth inhibition ratio of 95.1% (31).

**Figure 8:**
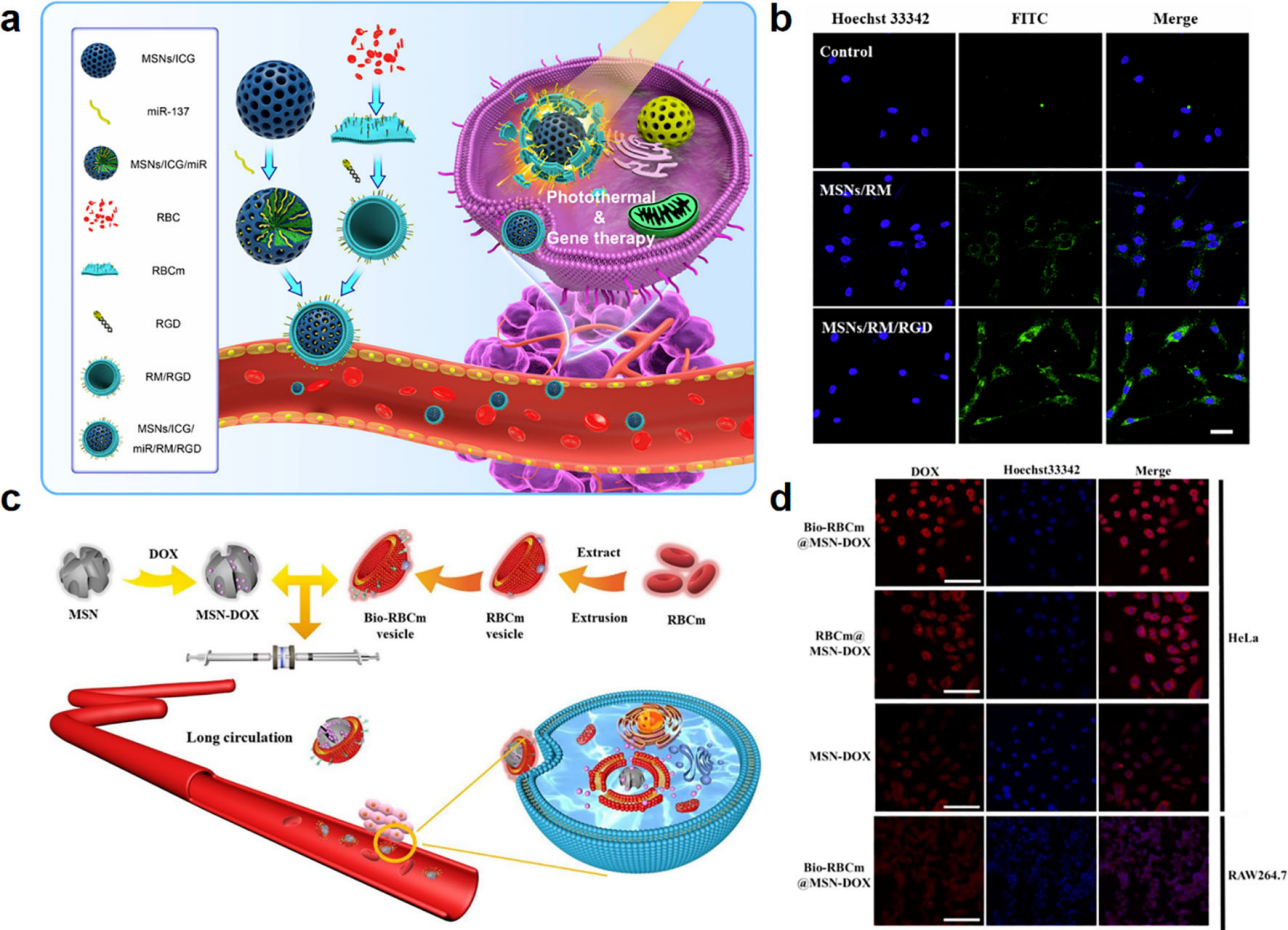
Em-MSNs for cancer therapy. **(a)** Schematic illustration of MSNs/ICG/miR/RM/RGD for tumor treatment and bio-imaging (32). **(b)** CLSM images of U87 cells after incubating with MSNs, MSNs/RM, and MSNs/RM/RGD (MSNs labeled with FITC for 4 h. Scale bar = 20 μm). Reproduced with permission (32). ^©^ 2022 Li et al. Nanomaterials published by MDPI AG. **(c)** Schematic illustration of the design of the Bio-RBCm@MSN–DOX biomimetic nanoparticle (33). **(d)** Targeting ability and anti-phagocytosis ability of Bio-RBCm@MSN–DOX, RBCm@MSN–DOX, MSN–DOX co-cultured with HeLa cells, and Bio-RBCm@MSN–DOX co-cultured with RAW264.7 cells. Scale bar = 50 μm. Reproduced with permission (33). ^©^ 2021 Zhang et al. New Journal of Chemistry published by The Royal Society of Chemistry.

RGD peptide can inhibit integrin-ligand interactions through binding to αVβ3 and αVβ5 integrins overexpressed in tumor vasculature (32). Thus, although the Em itself does not have the ability to actively target tumor site, Em modified with RGD peptide such as cRGD and iRGD could penetrate tumor blood vessels, thereby increasing the local drug concentrations in the tumor sites (177). Li et al. prepared cRGD modified Em, which was used as a coating for ICG and microRNAs-137 loaded MSNs. The tumor inhibition rate increased from 74.4% to 94.9% after cRGD peptide modification due to the long circulation ability of Em and the active targeting ability of cRGD (**Figures 8a, b**). Other molecules with cancer targeting ability can be also used for Em modification. For instance, biotin receptors are overexpressed in various types of solid tumors and play important roles in tumor metabolism, growth, and metastasis (178). Biotin modified Em-MSNs loaded with DOX (Bio-RBCm@MSN-DOX) exhibited enhanced cancer targeting and immune evasion due to the Em coating (**Figures 8c, d**) (33). Compared to bare MSNs, the ability of Bio-RBCm@MSN-DOX to target HeLa cells increased 4.64 folds, and the phagocytic efficiency of Bio-RBCm@MSN-DOX by macrophages decreased 2.55 folds. Moreover, DNA aptamer against mucin-1 (an important mucin glycoprotein-related marker expressed in human adenocarcinomas) was apply to modify Em, which further coated on the surface of DOX loaded hollow mesoporous organosilica nanoparticles (Apt-RBC-HMOS@DOX). In C26 tumor-bearing mice model, all mice stayed alive in Apt-RBC-HMOS@DOX group while all mice died in free DOX group in 30 days (34). Altogether, Em-MSNs that modified with active targeting ligands show long circulation and cancer targeting functions, providing a useful drug delivery system for the targeted therapy of cancers.

## 5 Prospective and challenges

Cell membrane coating have been widely used for surface modification of MSNs, which facilitate the interaction of MSNs with complex biological environment. This review summarizes recent advances in the rational design of CM-MSNs for anticancer therapy. It discusses the advantages of employing membranes derived from erythrocytes, platelets, immune cells, cancer cells, as well as hybrid membranes, and highlights their respective applications. The article emphasizes that the cell membrane coating confers properties including immune evasion, prolonged circulation, tumor vasculature adhesion, and TME targeting to the CM-MSNs platform. Concurrently, the synthetic MSN core provides the system with crucial capabilities for efficient drug loading and controlled release. The appropriate selection and combination of cell membrane and core material MSNs have the potential to provide more delicate designs for CM-MSN.

**Figure 9:**
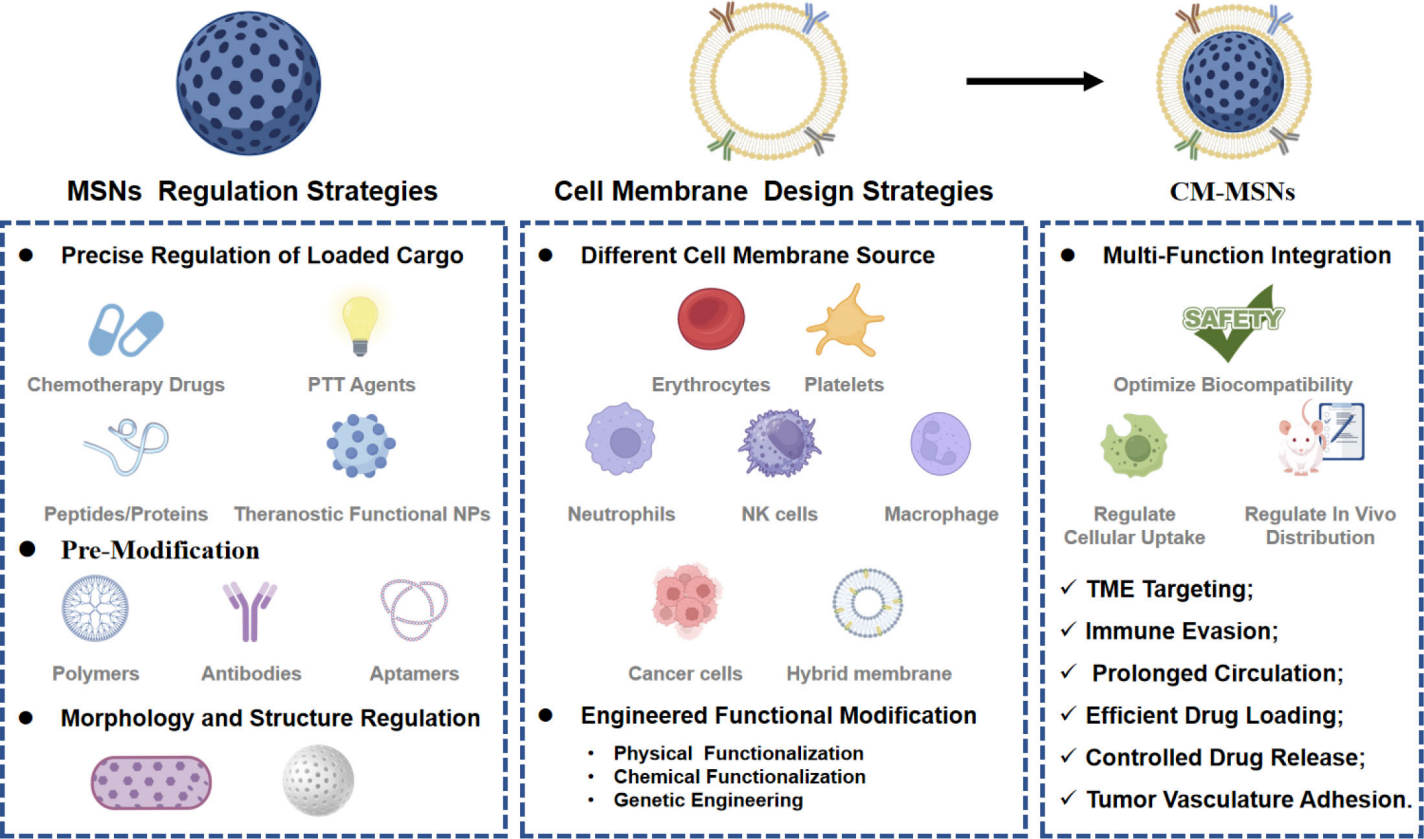
Different CM-MSNs platform design strategies.

The flexibility and stability of CM-MSNs are highly dependent on the design and performance of the MSN core and the source cell membrane. For the MSN core, tuning its physicochemical properties can introduce additional functions to meet specific application requirements, with three main approaches applied to optimize MSN performance. The most convenient method is adjusting the cargo loaded within MSNs, as the tunable porous structure of MSNs can encapsulate chemotherapy drugs, photosensitizers, photothermal agents, peptides, proteins, and even functional nanoparticles for cancer diagnosis and therapy. Pre-modification of MSNs with other functional materials before cell membrane coating is another common method, with an extremely wide range of optional materials including polymers, antibodies, aptamers and more. Tuning the morphology and appearance of MSNs also serves as an effective regulation method. For the cell membrane coating, targeted design strategies have been developed to expand the application of cell membrane coating technology in cancer treatment. So far, bio-membrane components from mammalian cells, bacteria, fungi, and even organelles, and the tremendous diversity of bio-membrane components extracted have been provided abundant flexible options for CM-MSN design. In addition, physical, chemical and genetic strategies for functionalizing cell membranes have further broadened the application scope of this biomimetic technology. With the rational design of the MSN core and cell membrane coating, the resulting CM-MSNs achieve excellent multi-function integration and exhibit a series of prominent advantages for cancer treatment, including optimized biocompatibility and safety, tunable cellular uptake, regulatable *in vivo* distribution, tumor microenvironment targeting, immune evasion, prolonged circulation time, efficient drug loading, controlled drug release, and favorable tumor vasculature adhesion (**Figure 9**).

However, numerous challenges remain in successfully translating CM-MSNs from bench to bedside. With the growing diversity in CM-MSN designs, there is an urgent need to establish rigorous standards to guarantee their efficacy and safety. These should encompass safety assurance of CM sources, compatibility with patient immune systems, and risk control during CM modification and MSN synthesis. Moreover, achieving large-scale manufacturing of CM-MSNs without compromising quality remains a major hurdle for clinical translation. The integration of natural biomaterials—endowed with biological properties—and synthetic materials with tunable physicochemical characteristics in the design of CM-coated MSN platforms will continue to drive the development of novel drug delivery systems for cancer therapy. Future research should focus on developing more sophisticated hybrid membrane technologies, for instance, by fusing membranes from different cell sources to integrate multiple functions. In parallel, in-depth proteomic analysis of membranes following *in vivo* circulation will identify changes in their composition. This strategy will facilitate the informed design of next-generation, bioinspired nanotherapeutics specifically engineered to modulate the tumor immune microenvironment and elicit robust anti-tumor effects.

## 6 Conclusion

This review highlights the promising potential of CM-MSNs in advancing cancer immunotherapy through precise modulation of the TIME. By integrating natural cell membranes with synthetic silica nanoparticles, CM-MSNs overcome key limitations of conventional nanocarriers. The biomimetic design not only provides passive immune evasion via membrane proteins such as CD47 but also facilitates active remodeling of the TIME through targeted immunomodulator delivery, immune cell reprogramming, and tumor antigen presentation. Although challenges in standardization and clinical translation remain, this work underscores the strategic role of CM-MSNs in bridging nanomedicine and immunotherapy. This nanotechnology-enabled immunomodulation represents a novel approach to overcoming resistance to existing immunotherapies and achieving synergistic efficacy, thereby establishing a solid foundation for developing next-generation, precision combination regimens for cancer treatment. next-generation, precision combination regimens for cancer treatment.

## Glossary

AAD: anti-angiogenic drug
BBB: blood-brain barrier
BMDMs: bone marrow-derived macrophages
BTB: blood-tumor barrier
CARs: chimeric antigen receptors
CCL2: chemokine (C-C motif) ligand 2
CCM: cancer cells membrane
CCR2: C-C chemokine receptor 2
CD200: cluster of differentiation 200
CD41: cluster of differentiation 41
CD44: cluster of differentiation 44
CD47: cluster of differentiation 47
CD61: cluster of differentiation 61
CD62p: cluster of differentiation 62P
CM: cell membrane
CM-MSNs: cell membrane-coated MSNs
CSF1: colony-stimulating factor 1
CSF1R: CSF1 receptor
CTCs: circulating tumor cells
CuS: copper sulfide
CXCL10: C-X-C motif chemokine ligand 10
CXCL9: C-X-C motif chemokine ligand 9
CXCR3: C-X-C motif chemokine receptor 3
DCs: dendritic cells
DOX: doxorubicin
Em: erythrocyte membrane
GPC3: Glypican-3
GPIa/IIa: glycoprotein Ia/IIa
GPIb: glycoprotein Ib
HCC: hepatocellular carcinoma
ICAM-1: intercellular adhesion molecule-1
ICAM-2: intercellular adhesion molecule-2
ICG: indocyanine green
IFN-γ: interferon-gamma
IL-10: interleukin-10
LFA-1: lymphocyte function-associated antigen-1
LPHM: leukocyte and platelet membranes
M1-MM: M1 macrophage membrane
MM: macrophage membranes
MSNs: Mesoporous silica nanoparticles
NIR: near-infrared
NK cells: natural killer cells
Nm: neutrophil cell membranes
PD - 1: programmed death – 1
PD - L1: programmed death - ligand 1
PDT: photodynamic therapy
PM: platelet membrane
PSGL-1: pregnancy-specific glycoprotein 1
PTT: photothermal therapy
RGD: arginine-glycine-aspartic acid
RM: RAW264.7 cell membrane
ScFv: single chain variable region
SIRPα: signal regulatory protein α
TA: tannic acid
TAMs: tumor-associated macrophages
TGF-β: transforming growth factor-beta
Th1 cells: T helper 1 cells
TIME: tumor immune microenvironment
TME: tumor microenvironment
TNBC: triple negative breast cancer
Tregs: regulatory T cells
VCAM-1: vascular cell adhesion molecule 1
VDAs: vascular disruption agents
VLA-4: variable light chain 1-4
αVβ3: integrin αvβ3
αVβ5: integrin αvβ5.
